# Real‐World Evaluation of Remimazolam for Sedation During Gastrointestinal Endoscopy: Efficacy, Safety, and Risk Factors

**DOI:** 10.1002/jgh3.70351

**Published:** 2026-02-16

**Authors:** Hinako Sakurai, Kurato Miyazaki, Atsushi Nakayama, Motoki Sasaki, Mai Oowada, Misaki Sugawara, Yuki Kubo, Rei Mizobe, Ai Katsumi, Maya Ishizawa, Yuri Imura, Shoma Murata, Daisuke Minezaki, Kentaro Iwata, Anna Tojo, Teppei Masunaga, Kumiko Kirita, Mari Mizutani, Michiko Nishikawa, Yusaku Takatori, Teppei Akimoto, Shintaro Kawasaki, Noriko Matsuura, Hideomi Tomida, Tomohisa Sujino, Kaoru Takabayashi, Kanai Takanori, Naohisa Yahagi, Motohiko Kato

**Affiliations:** ^1^ Division of Gastroenterology and Hepatology, Department of Internal Medicine Keio University School of Medicine Tokyo Japan; ^2^ Center for Diagnostic and Therapeutic Endoscopy Keio University School of Medicine Tokyo Japan; ^3^ Division of Research and Development for Minimally Invasive Treatment, Cancer Center Keio University School of Medicine Tokyo Japan; ^4^ Department of Gastroenterology Oita University Faculty of Medicine Oita Japan; ^5^ Division of Gastroenterology and Hepatology Saitama City Hospital Saitama Japan; ^6^ Department of Gastroenterological Oncology Hyogo Cancer Center Hyogo Japan; ^7^ Endoscopy Center Ehime University Hospital Ehime Japan; ^8^ Department of Multidimensional Analysis of Gastrointestinal Biology, Sakaguchi Laboratory Keio University School of Medicine Tokyo Japan; ^9^ Keio Global Research Institute Keio University Tokyo Japan

**Keywords:** benzodiazepines, drug‐related side effects and adverse reactions, gastrointestinal endoscopy, hypoxia, sedation

## Abstract

**Background and Aim:**

Remimazolam is a benzodiazepine receptor agonist intravenous anesthetic. This study aimed to evaluate the efficacy and safety of remimazolam for sedation during gastrointestinal endoscopy using real‐world clinical data.

**Methods:**

This retrospective observational study included 352 patients who underwent esophagogastroduodenoscopy or colonoscopy sedated with remimazolam between January and February 2024 at our institution. Outcomes included the incidence of awakening during procedures, the sedation completion rate, and the incidence and severity of adverse events. Multivariate logistic regression analyses identified factors associated with hypoxia and hypotension.

**Results:**

Median patient age was 67 years (IQR: 58–74), and 62.2% were male. Median initial and additional doses were 3 (IQR: 2–3 mg) and 1 mg (IQR: 0–2 mg). Awakening occurred in 19.0% of patients. The sedation completion rate was 100%. Adverse events included hypotension (7.8%), hypoxia (13.1%), and bradycardia (4.0%), and no serious adverse events were observed. The only risk factor for hypoxia was advanced age (Odds ratio 1.04, 95% confidence interval: 1.01–1.08, *p* = 0.03), and the dose of remimazolam itself was not an independent risk factor for either hypoxia or hypotension.

**Conclusions:**

Remimazolam usage for gastrointestinal endoscopic sedation showed a favorable safety profile. Advanced age was associated with an increased risk of hypoxia, suggesting that careful monitoring and individualized sedation protocols are especially necessary for elderly patients. On the other hand, there are still issues regarding the duration of sedation. During long procedures, it is necessary to frequently check the depth of sedation to avoid undersedation.

## 1 Introduction

Gastrointestinal endoscopy is a procedure associated with discomfort, and sedatives are commonly administered during the examination [1]. Traditionally, benzodiazepines have been the main sedatives for gastrointestinal endoscopy in Japan. Even midazolam, which is considered to be relatively rapidly metabolized, has a half‐life of 3–4 h and requires 30–60 min for recovery [2, 3, 4, 5].

Remimazolam is a benzodiazepine receptor agonist intravenous anesthetic that was first launched in Japan in August 2020. It is characterized by rapid onset and offset of action, owing to its distinct affinity for benzodiazepine binding sites, metabolic pathways, and the pharmacodynamic properties of its metabolites [3, 4, 5]. Its half‐life is 0.92 ± 0.16 h in healthy adults [6], approximately one‐third that of midazolam. It is metabolized in the liver by carboxylesterase into an inactive metabolite, which is predominantly (99%) excreted in the urine [7, 8]. Remimazolam offers superior advantages over other benzodiazepines in terms of the speed of procedural initiation and recovery, which may help reduce the workload of monitoring staff and increase procedural throughput in clinical settings [9, 10].

Its low incidence of adverse events has also garnered attention [11, 12, 13, 14, 15, 16, 17, 18, 19, 20, 21]. In a randomized controlled trial comparing the effects of remimazolam and propofol on oxygen reserve during upper gastrointestinal endoscopy, the decrease in oxygen reserve and the incidence of hypoxia were significantly lower in the remimazolam group [20].

In Japan, some clinical trials have investigated remimazolam for endoscopic sedation [2, 8, 22, 23, 24, 25, 26]. However, few facilities currently use remimazolam for endoscopic procedures, and to date, no real‐world data have been evaluated for its use in gastrointestinal endoscopy in Japan. The aim of this study was to evaluate the efficacy and safety of remimazolam as a novel sedative for gastrointestinal endoscopy using real‐world data in a patient population with less selection bias and closer to general clinical practice than in previously reported clinical trials.

## 2 Methods

### 2.1 Study Design

This study was a retrospective observational study. It was approved by the Institutional Review Board of Keio University Hospital (20180163) and conducted in compliance with the principles of the Declaration of Helsinki. Since it was limited to off‐label use at the time of its introduction, we applied to the hospital's Medical Safety Committee for approval of the off‐label use of remimazolam in gastrointestinal endoscopy and started using it in November 2023. Written informed consent was obtained from all patients who underwent endoscopy regarding the use of remimazolam and data extraction from medical records.

### 2.2 Patients

We analyzed patients who underwent pretreatment diagnostic or posttreatment surveillance endoscopy at our institution between January 2024 and February 2024. The procedures include esophagogastroduodenoscopy (EGD) and colonoscopy (CS). Patients who were not sedated with remimazolam or who underwent EGD and CS simultaneously were excluded.

### 2.3 Endoscopic Procedures and Sedation Protocol

All procedures were performed using an EVIS‐X1 endoscopy system (Olympus Medical Systems, Tokyo, Japan) or LASEREO7000 endoscopy system (FUJIFILM Corporation, Tokyo, Japan). All operators used a GIF‐XZ1200 scope (Olympus Medical Systems) or an EG‐L600ZW7 scope (FUJIFILM Corporation) for EGD, a PCF‐H290 ZI scope (Olympus Medical Systems) or an EC‐L600ZP7 scope (FUJIFILM Corporation) for CS.

The dosage and administration interval were determined based on the previously reported phase II investigator‐initiated clinical trial [2]. Remimazolam was injected at an initial dose of 3 mg. The initial dose was adjusted at the endoscopist's discretion, considering patient background and condition. The sedation level was assessed using the Modified Observer's Assessment of Alertness/Sedation (MOAA/S) score. The endoscopist aimed for a MOAA/S score of 4 or less, and if sedation was inadequate (MOAA/S score of 5), an additional dose of 1 mg of remimazolam was added. After the start of the procedure, an additional dose of 1 mg of remimazolam was administered at approximately 2–5 min intervals if sedation was inadequate. Unlike the previously reported clinical trial, we used pethidine 35 mg in combination and did not set an upper limit for the dosage of remimazolam in this study. If the sedative effect of remimazolam was inadequate to complete the procedure, the operator was allowed to administer other sedatives to the patient.

Vital signs, including blood pressure, pulse rate, and SpO_2_, were measured before procedures, during procedures at 5‐min intervals, and after procedures. Supplemental oxygen was provided if a decrease in SpO_2_ was observed. Extracellular fluid was administered if a decrease in blood pressure due to circulatory depression was observed.

After the procedure, flumazenil was injected as a remimazolam antagonist at the endoscopist's discretion.

### 2.4 Outcome Measures

All data were extracted from medical records. We evaluated the clinical characteristics of the study population, such as age, sex, and body surface area (BSA), comorbidities (respiratory, cardiovascular, hepatic, and renal diseases), and ASA‐PS (American Society of Anesthesiologists physical status). BSA was calculated using the Du Bois formula.

We also evaluated outcomes such as procedure time, initial, additional, and total dose of remimazolam, dose of pethidine, and administration of flumazenil. The dose administered prior to the start of the procedure was defined as the initial dose, and the dose added during the procedure was defined as the additional dose. Procedure time was defined as the interval between pressing the “start of examination” and “end of examination” buttons on the endoscopy system, as automatically recorded by the endoscopy system.

In addition, we evaluated outcomes related to sedation efficacy and safety. Efficacy was assessed by the incidence of awakening during procedure and the sedation completion rate. Awakening during procedure was evaluated based on the presence or absence of eye‐opening. During the procedure, the nurse monitored the patient and recorded whether the patient opened their eyes during the procedure in a standardized nursing chart. We defined sedation completion as the end of procedure without the need for additional administration of other sedatives. Safety was assessed by adverse events associated with sedatives such as postprocedural discomfort, poor awakening, hypotension, hypoxia, and bradycardia. Adverse events were investigated from the time of drug administration until the patient left the endoscopy room. We defined poor awakening as the inability to maintain eye opening in response to verbal stimulation. After the procedure, the nurse assessed and documented the level of the patient's awakening in a standardized nursing chart. We defined hypotension as systolic blood pressure less than 90 mmHg and at least 20% decrease from the pre‐endoscopy, hypoxia as SpO_2_ less than 90%, and bradycardia as pulse rate less than 50 per minute. The severity of adverse events was determined on the basis of the Common Terminology Criteria for Adverse Events version 5.0.

### 2.5 Statistical Analysis

Continuous variables are presented as median and interquartile range (IQR), and categorical variables are expressed as numbers and percentages. To evaluate differences between EGD and CS, continuous variables were compared using the two‐sided Mann–Whitney *U* test, and categorical variables were analyzed with Fisher's exact test. Univariate and multivariate logistic regression analyses were performed to identify factors associated with hypoxia and hypotension. The variables included in the univariate model were selected based on clinical relevance. The variables were considered candidates for multivariate modeling based on univariate screening results. Odds ratios (ORs) with 95% confidence intervals (CIs) were calculated. A two‐sided *p* < 0.05 was considered statistically significant. The total dose of remimazolam was compared among age groups using the Kruskal–Wallis test. To evaluate the linear relationship between increasing age and hypoxia incidence, Pearson's product–moment correlation coefficient (*r*) was calculated. Analyses were conducted using JMP (version 13.0.0, SAS Institute, Cary, NC, USA).

## 3 Results

### 3.1 Study Flowchart

**FIGURE 1:**
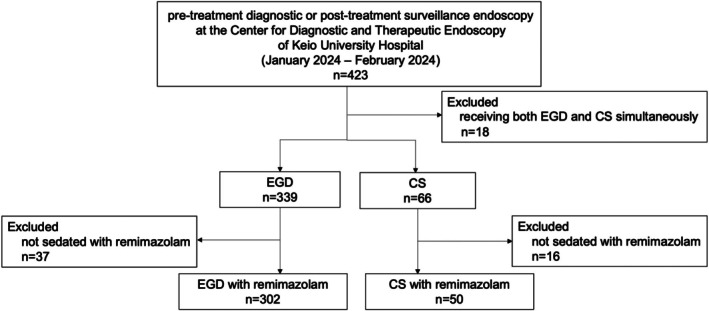
Study flowchart. Of 423 patients who underwent EGD or CS between January and February 2024, those receiving both EGD and CS (*n* = 18) were excluded. In the EGD cohort (*n* = 339), those not sedated with remimazolam (*n* = 37) were excluded. In the CS cohort (*n* = 66), those not sedated with remimazolam (*n* = 16) were excluded. Final analytic samples were EGD with remimazolam (*n* = 302) and CS with remimazolam (*n* = 50). CS, colonoscopy; EGD, esophagogastroduodenoscopy.

The flowchart of this study is presented in Figure 1. A total of 423 patients underwent pretreatment diagnostic or posttreatment surveillance endoscopy at our institution between January and February 2024, and patients who underwent both EGD and CS simultaneously (*n* = 18) and were not sedated with remimazolam (*n* = 37 for EGD; *n* = 16 for CS) were excluded. The reasons for not sedated with remimazolam included patient preference, hypotension prior to the procedure, history of hypersensitivity to benzodiazepines, and the operator's judgment that sedation is not appropriate. A total of 352 patients (*n* = 302 for EGD; *n* = 50 for CS) were included in the analysis.

### 3.2 Clinical Characteristics of Study Population

The characteristics of the patients are presented in Table 1. The median age was 67 years (IQR: 58–74 years), and 62.2% were male. The median BSA was 1.65 m^2^ (IQR: 1.51–1.78 m^2^).

**TABLE 1:** Clinical characteristics of the study population (*n* = 352).

		Overall (*n* = 352)	EGD (*n* = 302)	CS (*n* = 50)	*p*
Age	Median [IQR], (years)	67 [58–74]	67 [59–74]	66 [52–74]	0.19
Sex	Male, *n* (%)	219 (62.2%)	190 (62.9%)	29 (58.0%)	0.53
BSA	Median [IQR], (m^2^)	1.65 [1.51–1.78]	1.66 [1.52–1.78]	1.59 [1.48–1.74]	0.18
ASA‐PS	*n* (%)				0.54
1		60 (17.1%)	49 (16.2%)	11 (22.0%)	
2		247 (70.2%)	215 (71.2%)	32 (64.0%)	
3		45 (12.8%)	38 (12.6%)	7 (14.0%)	
4 or more		0 (0%)	0 (0%)	0 (0%)	
Respiratory disease	*n* (%)	43 (12.2%)	38 (12.6%)	5 (10.0%)	0.61
Cardiovascular disease	*n* (%)	45 (12.8%)	40 (13.3%)	5 (10.0%)	0.52
Hepatic disease	*n* (%)	20 (5.7%)	16 (5.3%)	4 (8.0%)	0.44
Renal disease	*n* (%)	23 (6.5%)	19 (6.3%)	4 (8.0%)	0.65

Abbreviations: ASA PS, American Society of Anesthesiologists physical status; BSA, body surface area; CS, colonoscopy; EGD, esophagogastroduodenoscopy; IQR, interquartile range.

In the overall cohort, most patients were classified as ASA‐PS II (70.2%), followed by ASA‐PS I (17.1%) and ASA‐PS III (12.8%); no patients had ASA‐PS IV or higher. Regarding comorbidities, respiratory disease was present in 43 patients (12.2%), cardiovascular disease in 45 (12.8%), hepatic disease in 20 (5.7%), and renal disease in 23 (6.5%).

No significant differences were observed in the characteristics of the patients between EGD and CS.

### 3.3 Procedure Outcomes

Procedure outcomes are summarized in Table 2. The median procedure time was 21.8 min (IQR: 15.9–30.8 min), with a significantly longer duration for CS compared to EGD (34.1 min [IQR: 21.0–43.3] vs. 21.5 min [IQR: 15.3–28.5]; *p* < 0.001). The median initial dose of remimazolam was 3 mg (IQR: 2–3 mg), and the median additional dose was 1 mg (IQR: 0–2 mg) overall. Twelve patients (3.4%) required additional administration prior to the start of the procedure and received the initial dose exceeding 3 mg. The median initial dose was significantly higher in EGD compared to CS (3 mg [IQR: 2–3 mg] vs. 2 mg [IQR: 2–3 mg]; *p* = 0.005). Similarly, the median additional dose was significantly higher in EGD compared to CS (1 mg [IQR: 0–2 mg] vs. 0 mg [IQR: 0–1 mg]; *p* = 0.02). Awakening during procedures occurred in 67 patients (19.0%). The sedation completion rate was 100%. Flumazenil was administered to 35 patients (9.9%). Among the 35 patients who received flumazenil, the documented reasons for administration were prophylactic reversal (*n* = 12), hypotension (*n* = 11), poor awakening (*n* = 9), discomfort (*n* = 2), and hypoxia (*n* = 1).

**TABLE 2:** Procedure outcomes (*n* = 352).

		Overall (*n* = 352)	EGD (*n* = 302)	CS (*n* = 50)	*p*
Procedure time	Median [IQR], min	21.8 [15.9–30.8]	21.5 [15.3–28.5]	34.1 [21.0–43.3]	< 0.001
Initial dose of remimazolam	Median [IQR], mg	3 [2–3]	3 [2–3]	2 [2–3]	0.005
Additional dose of remimazolam	Median [IQR], mg	1 [0–2]	1 [0–2]	0 [0–1]	0.02
Total dose of remimazolam	Median [IQR], mg	3 [2–4]	3 [2–4]	2.5 [2–4]	0.0025
Total dose of pethidine	Median [IQR], mg	35 [35–35]	35 [35–35]	35 [35–35]	0.79
Awakening during procedure	*n* (%)	67 (19.0%)	59 (19.5%)	8 (16.0%)	0.70
Sedation completion	*n* (%)	352 (100%)	302 (100%)	50 (100%)	1.00
Flumazenil administration	*n* (%)	35 (9.9%)	30 (9.9%)	5 (10.0%)	1.00

Abbreviations: CS, colonoscopy; EGD, esophagogastroduodenoscopy; IQR, interquartile range.

### 3.4 Adverse Events of Sedation

Sedation‐related adverse events are summarized in Table 3. Poor awakening after the procedure was documented in nine patients (2.6%), and discomfort after the procedure was reported in eight patients (2.3%). Hypotension occurred in 27 patients (7.8%), with Grade 1 observed in 18 patients (5.1%) and Grade 2 in nine patients (2.6%). No patients experienced Grade 3 or higher hypotension. Hypoxia was the most common adverse event and observed in 46 patients (13.1%). Grade 1 hypoxia occurred in 19 patients (5.4%), while Grade 2 hypoxia was seen in 27 patients (7.7%). There were no cases of Grade 3 or higher hypoxia. Bradycardia was reported in 13 patients (4.0%), all of which were Grade 1. There were no Grade 2 or higher bradycardia events.

**TABLE 3:** Adverse events of sedation.

		Overall (*n* = 352)	EGD (*n* = 302)	CS (*n* = 50)	*p*
Poor awakening after procedure	*n* (%)	9 (2.6%)	7 (2.3%)	2 (4.0%)	0.62
Discomfort after procedure	*n* (%)	8 (2.3%)	7 (2.3%)	1 (2.0%)	1.00
Hypotension	*n* (%)	27 (7.8%)	22 (7.4%)	5 (10.0%)	1.00
Grade 1		18 (5.1%)	14 (4.6%)	4 (8.0%)	
Grade 2		9 (2.6%)	8 (2.6%)	1 (2.0%)	
Grade 3 or more		0 (0%)	0 (0%)	0 (0%)	
Hypoxia	*n* (%)	46 (13.1%)	42 (14.0%)	4 (8.0%)	0.36
Grade 1		19 (5.4%)	18 (6.0%)	1 (2.0%)	
Grade 2		27 (7.7%)	24 (7.9%)	3 (6.0%)	
Grade 3 or more		0 (0%)	0 (0%)	0 (0%)	
Bradycardia	*n* (%)	13 (4.0%)	13 (4.6%)	0 (0%)	0.23
Grade 1		13 (4.0%)	13 (4.6%)	0 (0%)	
Grade 2		0 (0%)	0 (0%)	0 (0%)	
Grade 3 or more		0 (0%)	0 (0%)	0 (0%)	

*Note:* Adverse events were graded according to the Common Terminology Criteria for Adverse Events version 5.0.

Abbreviations: CS, colonoscopy; EGD, esophagogastroduodenoscopy.

These adverse events were generally manageable and reversible with appropriate supportive care, such as fluid resuscitation, oxygen administration, or administration of reversal agents when necessary. No significant differences were observed between EGD and CS in the incidence of each adverse event.

### 3.5 Risk Factors of Hypoxia

Univariate logistic regression analyses were performed to evaluate risk factors associated with hypoxia using age, BSA, type of procedure, total dose of remimazolam, total dose of pethidine, and ASA‐PS score as risk factors (Table 4). The results showed that age was the only significant risk factor (OR: 1.03, 95% CI: 1.00–1.06, *p* = 0.05). Next, five covariates were selected based on univariate screening results: age, type of procedure, total dose of remimazolam, total dose of pethidine, and ASA‐PS score to perform multivariate logistic regression analyses, and age was identified as a significant risk factor as well (OR: 1.04, 95% CI: 1.01–1.08, *p* = 0.03), suggesting that older patients were at greater risk of developing hypoxic events during sedation. Other factors were not significantly associated with hypoxia.

**TABLE 4:** Risk factors causing hypoxia.

Factors	Univariate analysis		Multivariate analysis	
	OR [95% CI]	*p*	OR [95% CI]	*p*
Age (per 1 year)	1.03 [1.00–1.06]	0.05	1.04 [1.01–1.08]	0.03
Procedure				
EGD	1.86 [0.64–5.45]	0.25	1.54 [0.57–5.43]	0.42
CS	1		1	
Total dose of remimazolam (per 1 mg)	1.08 [0.89–1.32]	0.43	1.13 [0.89–1.41]	0.31
Total dose of pethidine (per 10 mg)	1.08 [0.82–1.40]	0.59	1.17 [0.86–1.60]	0.33
ASA‐PS (per 1 point)	1.42 [0.80–2.54]	0.23	1.17 [0.62–2.21]	0.62
BSA (per 0.1 m^2^)	1.00 [0.83–1.21]	0.98		

Abbreviations: ASA PS, American Society of Anesthesiologists physical status; BSA, body surface area; CI, confidence interval; CS, colonoscopy; EGD, esophagogastroduodenoscopy; OR, odds ratio.

### 3.6 Risk Factors of Hypotension

Univariate logistic regression analyses were performed to evaluate risk factors associated with hypotension using age, BSA, type of procedure, total dose of remimazolam, total dose of pethidine, and ASA‐PS score as risk factors (Table 5). The results showed that none of these factors were significant risk factors. Next, two covariates were selected based on univariate screening results: total dose of remimazolam and BSA to perform multivariate logistic regression analyses; however, none of these variables showed a statistically significant association with hypotension (all *p* ≥ 0.30).

**TABLE 5:** Risk factors causing hypotension.

Factors	Univariate analysis		Multivariate analysis	
	OR [95% CI]	*p*	OR [95% CI]	*p*
Age (per 1 year)	1.00 [0.97–1.04]	0.81		
Procedure				
EGD	0.96 [0.32–2.91]	0.95		
CS	1			
Total dose of remimazolam (per 1 mg)	0.82 [0.61–1.10]	0.16	0.85 [0.61–1.14]	0.30
Total dose of pethidine (per 10 mg)	0.92 [0.65–1.29]	0.62		
ASA‐PS (per 1 point)	0.91 [0.44–1.88]	0.80		
BSA (per 0.1 m^2^)	0.88 [0.69–1.12]	0.31	0.35 [0.03–3.75]	0.39

Abbreviations: ASA PS, American Society of Anesthesiologists physical status; BSA, body surface area; CI, confidence interval; CS, colonoscopy; EGD, esophagogastroduodenoscopy; OR, odds ratio.

### 3.7 Incidence of Hypoxia by Age Group

**FIGURE 2:**
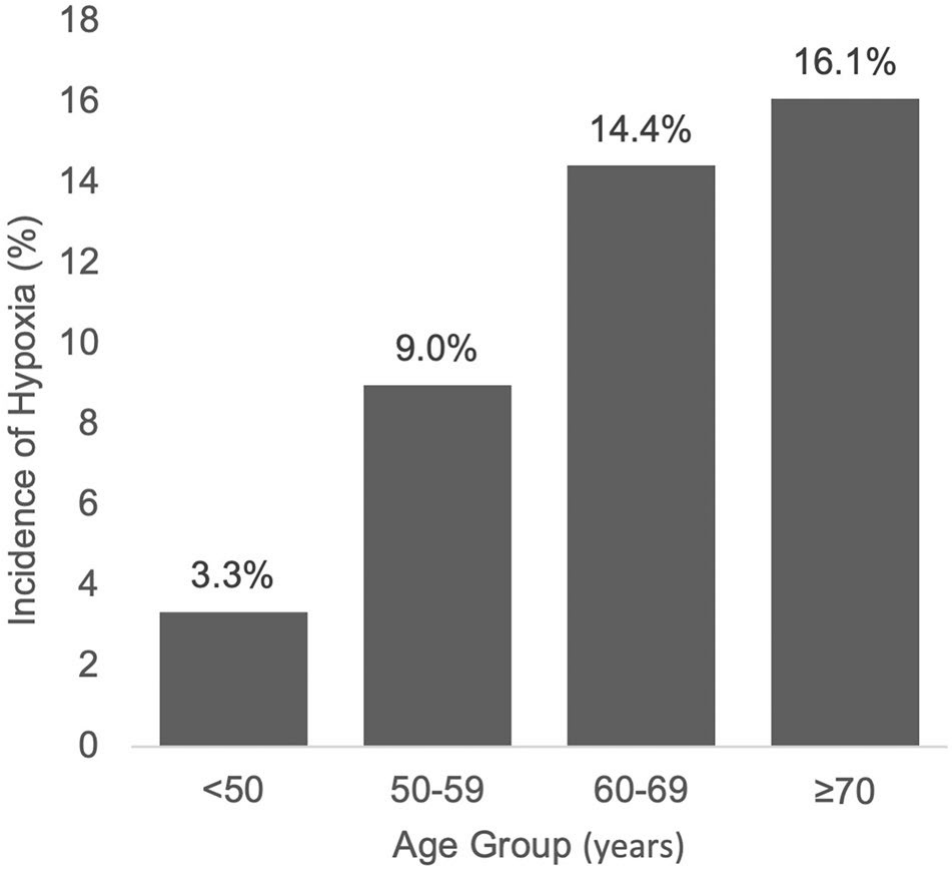
Incidence of hypoxia (SpO_2_ < 90%) stratified by age group (< 50, 50–59, 60–69, ≥ 70 years). Dotted line indicates linear fit. Rates increased with age: 3.3%, 9.0%, 14.4%, and 16.1%, respectively.

The incidence of hypoxia was evaluated across four age categories: < 50, 50–59, 60–69, and ≥ 70 years. Hypoxia occurred in 3.3% of patients aged < 50 years, 9.0% of those aged 50–59 years, 14.4% of those aged 60–69 years, and 16.1% of those aged ≥ 70 years (Figure 2). There was a clear trend of increasing hypoxia incidence with age, with the highest rate observed in the ≥ 70 age group.

Treating age as a continuous variable and occurrence of hypoxia as the dependent variable, Pearson's product–moment correlation demonstrated a modest positive association between age and occurrence of hypoxia (*r* = 0.105, *p* = 0.05).

We additionally compared the total dose of remimazolam among age groups (Table 6). The median total doses of remimazolam in patients aged < 50, 50–59, 60–69, and ≥ 70 years were 4 mg (IQR: 3–5 mg), 4 mg (IQR: 3–4 mg), 3 mg (IQR: 3–5 mg), and 3 mg (IQR: 2–3 mg), respectively. The total dose of remimazolam was significantly lower in older patients (*p* < 0.001).

**TABLE 6:** Total dose of remimazolam according to age group.

	*n*	Total dose of remimazolam median [IQR], mg	*p*
< 50	30	4 [3–5]	< 0.001
50–59	67	4 [3–4]	
60–69	112	3 [3–5]	
≥ 70	143	3 [2–3]	

Abbreviation: IQR, interquartile range.

## 4 Discussion

In this study, we examined the efficacy and safety of remimazolam for endoscopic sedation in Japan using real‐world data. The overall incidence of adverse events was low, with most events being mild and reversible. Multivariate analysis identified advanced age as a significant risk factor for hypoxia. On the other hand, the frequency of awakening during procedures was relatively high.

When looking at the types of endoscopes, although baseline characteristics were comparable, EGD had a shorter procedure time yet required higher remimazolam doses than CS. This likely reflects greater discomfort due to gag reflex during EGD, prompting operators to target deeper sedation. By contrast, unless the patient complains of severe pain, operators can usually proceed with CS under relatively light sedation; accordingly, sedation depth may have been checked less often and supplemental dosing given less frequently. Thus, in real‐world CS, sedation tends to be shallow, warranting caution and regular reassessment to avoid undersedation.

With regard to adverse events, the low incidence and predominantly minor nature of adverse events observed in this study suggest a favorable safety profile for remimazolam. Importantly, the dose of remimazolam itself was not an independent risk factor for hypoxia and hypotension. The minimal adverse effects of remimazolam on respiration have been previously reported [20]. Moreover, owing to its short elimination half‐life and rapid metabolism by tissue esterases, remimazolam is unlikely to accumulate even when administered repeatedly as small boluses, as performed in this study. Therefore, the total administered dose may not accurately reflect clinically relevant drug exposure and this pharmacokinetic characteristic is considered to be the reason why the total dose was not an independent risk factor for adverse events. In contrast, advanced age was identified as an independent risk factor for hypoxia, likely due to increased drug sensitivity and reduced respiratory reserve in elderly patients. Comparing the incidence of hypoxia by age group, there was a clear trend of increasing occurrence of hypoxia with age, with the highest rate observed in the ≥ 70 age group though the total dose of remimazolam was significantly lower in older patients. Therefore, particularly for elderly patients aged 70 years or older, who are at higher risk of respiratory depression, careful management is required in clinical practice, such as strengthening monitoring and setting a low threshold for oxygen administration. Individualizing sedation protocols according to patient conditions is important to maximizing both safety and comfort.

In Japan, some clinical trials have investigated remimazolam for endoscopic sedation [2, 8, 22, 23, 24, 25, 26]. In a Phase II dose‐finding study by Ichijima et al., an initial dose of 3 mg followed by 1 mg supplements was reported to be both effective and safe [2], consistent with our findings. In the Phase III trial, remimazolam demonstrated a significantly higher sedation success rate compared to placebo (91.9%–95%) and fast recovery times. Reported adverse event rates included hypotension in 0%–5.6% of patients and hypoxia or requirement for supplemental oxygen in 2.8%–5% of patients [24, 26]. Since patients with background diseases are excluded and the average age of patients is lower in the previously reported clinical trials, the relatively higher rates of adverse events in our study may be attributed to the diverse clinical backgrounds of patients in a real‐world setting, highlighting the challenges of sedation management in actual clinical practice compared to controlled trial conditions. In terms of the efficacy of remimazolam, awakening during procedures occurred in 19.0% of patients in this study, and challenges remain regarding the persistence of sedation. This may be due to the longer procedure time in this study than in the previously reported clinical trial. Because of the variability in procedure time, the dose of remimazolam in clinical trials may not be sufficient in a real‐world setting. If the procedure time is prolonged, it is necessary to check the depth of sedation regularly to avoid undersedation.

This study has several advantages. First, to our knowledge, this is the first real‐world evaluation and the largest‐scale study of remimazolam for gastrointestinal endoscopic sedation in Japan. Second, we did not use age or weight as exclusion criteria for patients in this study. Third, we collected data on both EGD and CS, which account for the majority of gastrointestinal endoscopy. We provided data from 352 patients undergoing EGD and CS without exclusion based on age or weight, capturing diverse clinical backgrounds and administration practices. The use of remimazolam is expected to increase further in the future, and this will serve as important evidence in advance of that.

This study also has several limitations. First, its single‐center, retrospective observational design introduces potential selection bias. Second, it is a retrospective study and the data which can be extracted from medical records are limited. The data on concomitant medications and patient satisfaction were not evaluated in this study. Concomitant medications such as chronic use of opioids or benzodiazepines may have influenced the incidence of hypoxia and hypotension, and therefore the observed rates of these adverse events should be interpreted with caution. Third, since remimazolam and pethidine were used in combination in this study, the adverse event outcomes cannot be attributed solely to remimazolam. Fourth, the dosage and timing of remimazolam, oxygen, extracellular fluid and flumazenil administration were left to operator discretion, resulting in inconsistency. Finally, although we adjusted for major covariates in multivariable logistic regression, residual confounding by measured and unmeasured factors cannot be completely excluded and may have influenced the observed incidence of adverse events.

This study demonstrated a favorable safety profile of remimazolam. Remimazolam can be used as a safe sedative option for gastrointestinal endoscopy, but careful management is required for elderly patients aged 70 years or older, who are at higher risk of respiratory depression. On the other hand, the frequency of awakenings during procedures was relatively high, and issues remained regarding its effectiveness. Because remimazolam has a short duration of action, it is necessary to frequently check the depth of sedation and to administer an additional dose in order to avoid undersedation during long procedures. Regarding this issue, continuous intravenous infusion of remimazolam may be one solution. Further trials comparing continuous infusion with intermittent boluses are warranted.

## Funding

Research grant was provided to author M.K. by Mundipharma Co., Ltd.; however, it was not used for this study.

## Ethics Statement

This retrospective observational study was approved by the Institutional Review Board of Keio University Hospital (Approval No. 20180163) and conducted in accordance with the principles of the Declaration of Helsinki. Written informed consent was obtained from all patients for the use of remimazolam during endoscopy and for data extraction from medical records.

## Conflicts of Interest

M.K. has received a research grant and lecture honoraria from Mundipharma Co. Ltd. The other authors declare no conflicts of interest.

## Data Availability

The data that support the findings of this study are available on request from the corresponding author. The data are not publicly available due to privacy or ethical restrictions.
